# Catch up with authors across the history of Biology Open

**DOI:** 10.1242/bio.062200

**Published:** 2025-09-17

**Authors:** Saanjbati Adhikari

**Affiliations:** Cross-title Features Editor, The Company of Biologists, UK


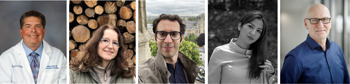


**Contributors to this article**. Left to right: Michael Herbert, Albena Dinkova-Kostova, Andrea Paterlini, Daisy Pineda-Suazo, Wim van der Poel.

The Company of Biologists is celebrating its 100-year anniversary in 2025. As a not-for-profit publisher run by distinguished practicing scientists, we publish five specialist high-quality journals: Development, Journal of Cell Science, Journal of Experimental Biology, Disease Models & Mechanisms (DMM) and Biology Open (BiO).

The first issue of BiO was published in 2012 with the primary objective of publishing “good-quality, sound research, without attempting to judge impact or novelty. It will be up to the scientific community to decide, after publication, on the importance of each paper”, shared Jordan Raff (University of Oxford, UK), BiO's inaugural Editor-in-Chief (EiC) (Raff, 2012). Over the past 13 years, BiO has created a unique platform for authors across different biological disciplines, including communities catered by BiO's sister journals – Development, Journal of Cell Science, Journal of Experimental Biology and DMM – to submit their research.

For this Editorial, I spoke with five authors of impactful research papers and review-type articles in BiO, some of whom have also been regular contributors to the journal. I asked them about their initial impressions of BiO, their experiences with the peer-review process, their favourite aspects of publishing with the journal and how publishing in BiO may have impacted their careers.

## Publishing in BiO for the first time

To set the stage, I reached out to some of our authors to learn how they first discovered BiO and what motivated them to publish with us. For Michael Hebert (University of Mississippi Medical Center, USA), the decision was driven by his admiration for the mission and initiatives of our publisher, The Company of Biologists. Hebert said, “I have always been a supporter of The Company of Biologists, because I am not an advocate of publishing in journals solely based on impact factor. Consequently, when BiO was launched, I was enthusiastic about supporting its mission to evaluate scientific work based on their impact within the community, rather than relying solely on individual or combined metrics”. Complimenting the integrity of the publication process in BiO, Hebert went on to say that “rapid, rigorous and fair review under the auspices of the Company, which holds a good reputation in the life science community” also made BiO an attractive option. Since publishing his first article as a corresponding author in BiO in 2013 (Broome et al., 2013), Hebert has published ten articles in the journal. Hebert also supports BiO's easy transfer option, which allows manuscripts submitted to other journals from The Company of Biologists to be transferred to BiO pre- or post-peer review.

It was the BiO transfer network that enabled Albena Dinkova-Kostova (University of Dundee, UK; Box 1) to publish in BiO. “When we submitted the paper to Journal of Cell Science, the Editors recommended transferring it to BiO – a then-new journal that focused on scientific rigour and sound methodology, rather than emphasising wide appeal. We were confident about the quality of our work, and we also strongly believed in its far-reaching potential. Most of all, we wished to share our findings with the scientific community as soon as possible, facilitating the development of the young investigators, Kira Holmström and Liam Baird, towards further discoveries”, said Dinkova-Kostova. When asked to share why BiO appealed as a publication venue, she replied, “When we submitted our manuscript to BiO, the journal had no impact factor because it was newly founded – much like our work. Previously, no one had considered that the transcription factor nuclear factor erythroid 2-related factor 2 (NRF2) may have a role in mitochondrial function, which we showed in our study. New ideas and discoveries are often unappealing to conventional thinkers and are met with scepticism”. Dinkova-Kostova's corresponding author paper published in BiO is now the most-cited article published in the journal to date (Holmström et al., 2013).

Box 1. Summary of the authors' BiO papers **Albena Dinkova-Kostova:** Our work was a collaboration with Andrey Abramov (University College London, UK) (Holmström et al., 2013). It tested the hypothesis that the transcription factor NRF2 affects bioenergetics and provided the foundation for further studies on this aspect of NRF2 biology. Prior to this study, NRF2 was known to orchestrate an elaborate genetic program that ensures cytoprotection, including drug metabolism, antioxidant defences and anti-inflammatory networks. Our study added another cytoprotective mechanism to the repertoire of NRF2 functions by demonstrating its profound influence on energy metabolism, thus supporting the usefulness of NRF2 activation in a wide range of pathological conditions and inspiring the Reata Pharmaceuticals-led clinical development of the NRF2 activator omaveloxolone (Skyclarys™) for Friedreich's ataxia patients.

**Andrea Paterlini:** The theme of my Review (Paterlini, 2020) organically emerged from what I was working on at the time: the regulation of shoot branching in *Arabidopsis thaliana*. Plant hormones were known to play key roles in the process but the relevance of their transport via plasmodesmata and phloem vascular tissues was less defined.

**Daisy Pineda-Suazo:** Our study provides the first detailed characterisation of digestive enzyme activity in *Octopus maya*, demonstrating the predominant role of cathepsins B, H and L in protein digestion and identifying key differences in enzymatic activation energy between acidic and alkaline environments (Pineda-Suazo et al., 2024).

**Wim van der Poel:** We showed that bees can detect SARS-CoV-2-infected clinical samples (Kontos et al., 2022). Apparently, volatiles induced by SARS-CoV-2 infection in humans and mammals enable bees to discriminate between infected and non-infected specimen. This is an interesting observation because it opens options for the development of SARS-CoV-2 diagnostics without the need for invasive sampling.

**Fig. 1.**
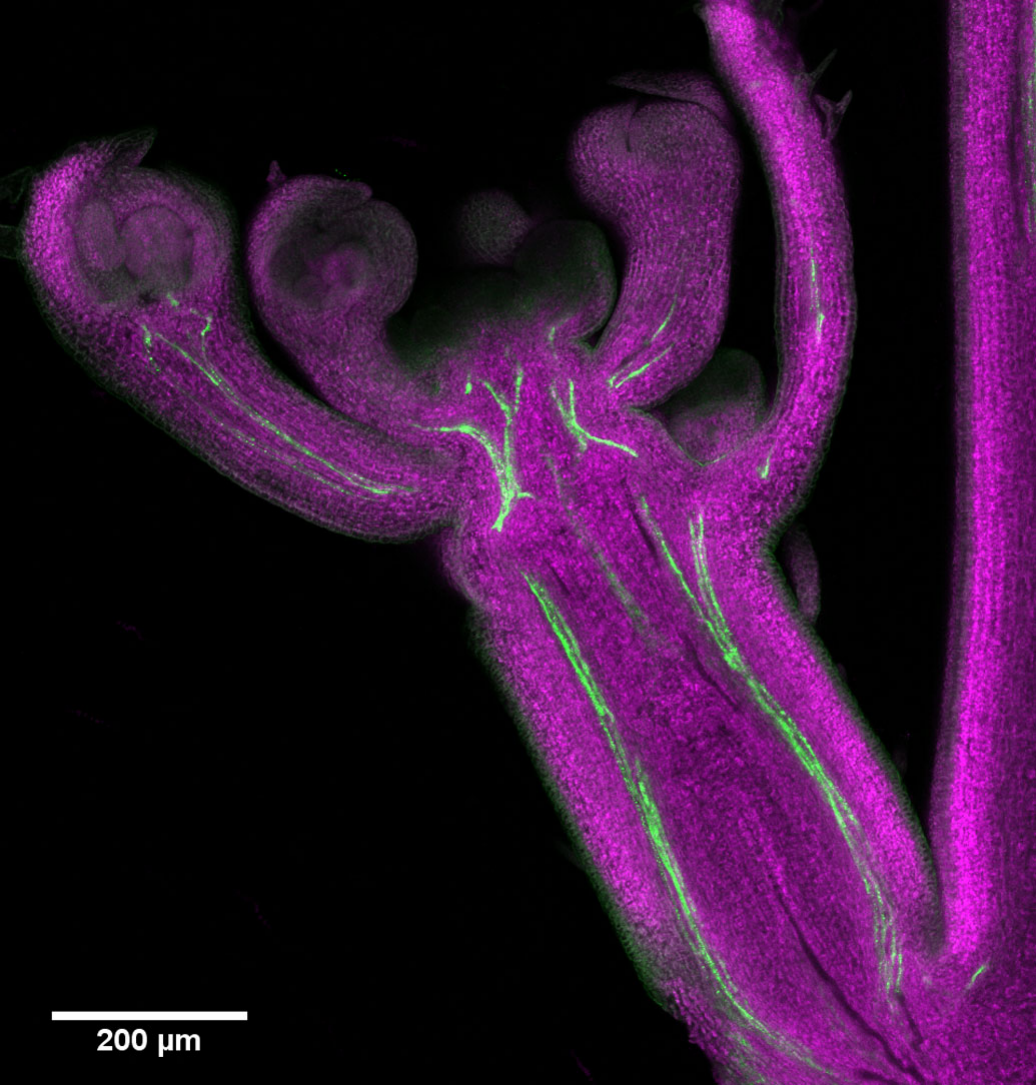
**Longitudinal section across an axillary bud of the *Arabidopsis thaliana* inflorescence.** Buds, upon activation, can grow out into shoot branches. In the confocal microscopy image chlorophyll autofluorescence is false coloured in magenta while the fluorescence signal of a phloem marker is rendered in green. Plasmodesmata in the phloem might be important for cell-cell and long-distance transport of specific factors, including plant hormones, that could control the bud activation process.

BiO's First Person interview series features first authors of a selection of papers published in BiO, helping early-career researchers (ECRs) promote themselves alongside their papers. Andrea Paterlini (University of Edinburgh, UK; Box 1, Fig. 1) published a Review in BiO in 2020 (Paterlini, 2020) as a part of our Future Leader Reviews series, which allows ECRs to independently publish Review articles in BiO. In recognition of his work, Paterlini was also the first ECR to be highlighted within the Future Leaders to Watch subsection of the First Person interview series. When asked how he first came across BiO, Paterlini shared, “I first heard of the Future Leader Reviews initiative on academic Twitter (now X). I was just finishing my PhD at the time, and I jumped at the opportunity because showcasing your thinking and gaining some visibility can be very valuable for ECRs”.

Another ECR, Daisy Pineda-Suazo (Universidad Nacional Autónoma de México, Mexico) was recently interviewed for our First Person series after her first-author publication in BiO (Pineda-Suazo et al., 2024; Box 1). Pineda-Suazo became aware of BiO through her peers. “My colleagues, who had previously published in the journal, recommended BiO for its rigorous but supportive peer-review process. Additionally, its Open Access (OA) format and focus on high-quality biological research made it an attractive option for disseminating my findings to a broader scientific audience”, shared Pineda-Suazo.

**Fig. 2.**
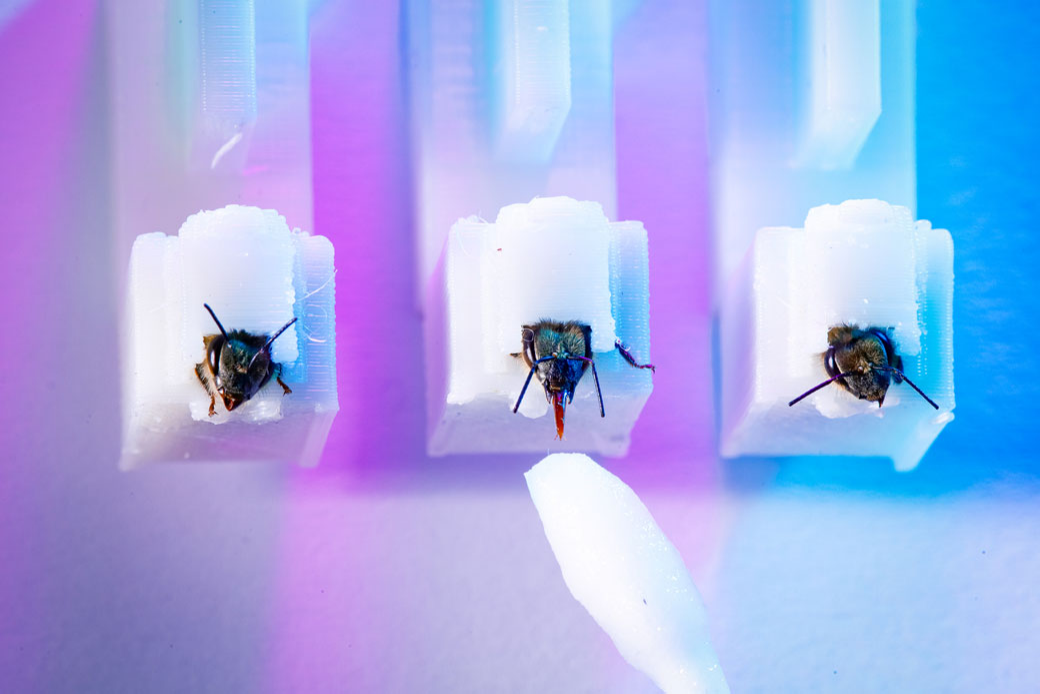
**Honeybees (** ***Apis mellifera*** **) used to identify SARS-CoV-2 infected minks (Neovison vison) in Kontos et al. (2022).**

One of BiO's founding aims was to publish research ‘across the breadth of the biological and biomedical sciences’, which includes animal physiology and disease biology. Wim van der Poel's (European College of Veterinary Microbiology, the Netherlands) BiO article on how bees can be trained to detect samples infected with a strain of coronavirus (Kontos et al., 2022; Box 1, Fig. 2) received notable media attention. Van der Poel told me that he had initially chosen Journal of Experimental Biology to publish this work “because of the involvement of bees and the experimental character of the study. We then were advised to submit to BiO,” said Van der Poel, “which we did because our main aim was to publish our manuscript in an OA journal that publishes research for a broad scientific community across biomedical sciences”.

Publishing with BiO was so smooth and rewarding that I decided to go through the entire process a second time

## The author experience at BiO

At The Company of Biologists (and thereby at BiO), we give utmost importance to independent, unbiased and critical assessment of scholarly publications. Similar to our sister journals, we also rely heavily on the expertise and experience of our Editors, Editorial Board and peer reviewers to regulate the quality of the research published in BiO. Furthermore, our dedicated in-house team ensure that all important steps – from manuscript submission to peer review – are handled smoothly (Adhikari et al., 2025).

When asked to share his firsthand experience of the peer review process at BiO, Hebert said that “it is conducted in a timely and rigorous manner”. He also elaborated on BiO's publication process, saying, “The Editors do a great job in ensuring that the paper is quickly sent out to re-review if necessary. After acceptance, the Production Editors make sure that all data meet the standards of the journal and are very professional. Proofs arrive quickly after integrity signs-offs and the paper is published”. Along similar lines, Dinkova-Kostova described her experience of the peer review process at BiO as “exactly what it was meant to be: tough, but fair”.

After his first publication in BiO as an ECR, Paterlini wrote another Review for our ‘A Year at the Forefront of…’ series (which aims to highlight the key discoveries and innovations that have recently made an impact in a specific biological field) as an independent group leader. Cherishing his growing relationship with the journal, Paterlini said, “Publishing with BiO was so smooth and rewarding that I decided to go through the entire process a second time. I recently wrote a Review (Paterlini, 2023), which was a helpful opportunity because I had just secured an independent position at the time. Surveying then-recent developments in my field was a valuable exercise to start planning my own (future) contributions”.

On another note, Van der Poel highlighted that the submission process in BiO was conducted very efficiently, along with a constructive peer review process. He went on to share, “BiO was also helpful in bringing our paper to the attention of a broad audience via social media, which resulted in a lot of media attention internationally. In conclusion, our experience with BiO was very positive. We hope that the journal continues to accommodate swift submission of manuscripts as well as fast and constructive reviewing in biological research”.

The First Person interview allowed me to share my research journey, highlighting the challenges and innovations involved in studying *O. maya* digestion

## BiO's commitment to ECRs

Supporting ECRs has always been a Company priority, and so we try to shine a light on the next generation of researchers at BiO. As an ECR herself, Pineda-Suazo noted that “BiO stands out for its commitment to promoting ECRs by providing a platform that ensures accessibility and visibility of their work”. She further praised the journal's “efficient editorial process, constructive peer review and OA policy” – all of which, she says, “fosters a collaborative and inclusive research environment, making it an excellent venue for publishing innovative biological studies”. Publishing her first-author paper in BiO significantly enhanced the visibility of Pineda-Suazo's research in the scientific community, particularly within the communities of comparative physiology and digestive biochemistry. She elaborated on her experience, saying, “The First Person interview allowed me to share my research journey, highlighting the challenges and innovations involved in studying *O. maya* digestion. This exposure facilitated new collaborations and strengthened my position for postdoctoral opportunities, helping me further develop my career in aquatic animal nutrition and physiology”.

Along similar lines, Paterlini, who initially published with us as an ECR and then again as a principal investigator said, “My Review has been well received by the community, having now accumulated more citations than some of my primary research papers”. At BiO, we publish front-section content (including Editorials, review-type pieces and interviews), covering relevant scientific topics that are of interest to our broad readership as well as educational pieces on scientific research and publishing trends. Reflecting on the impact of his 2020 BiO Review, Paterlini told me, “It [the Review article] was perhaps timely because several studies have since further explored the topic. Being the sole author of the article, as well as being featured in an associated interview, were helpful confidence boosts I returned to (for comfort) during more challenging research times”.

## Concluding remarks

These responses collectively highlight how BiO continues to fulfil the vision set forth by former EiCs – Jordan Raff and Steven Kelly (University of Oxford, UK) – and upheld by current EiC, Daniel Gorelick (Baylor College of Medicine, USA). Recently, BiO launched an ambitious new initiative: the Fast & Fair peer review process, described as ‘an experiment designed to deliver rigorous, high-quality peer review within seven business days’ (Gorelick and Clark, 2025). Although Paterlini hasn't experienced the new model yet, he views it as “a valuable approach that is worth exploring”. He further went on to say, “Recognising reviewers’ time and re-adjusting the distribution of benefits in the publishing system are positive moves. Defined timelines for publications are also highly beneficial for authors”. Sharing a similar viewpoint, Van der Poel said, “I think this is a very good initiative, and we agree that timely and transparent reviewing is very important”.

As BiO continues to innovate, I look forward to reconnecting with more authors in the coming years to reflect on the impact of this latest initiative and to discover what new developments have emerged to further support biologists in sharing their science with the community.
